# Supplementary material for: Addressing challenges with real-world synthetic control arms to demonstrate the comparative effectiveness of Pralsetinib in non-small cell lung cancer
Source: Nat Commun. 2022 Jun 17;13:3500. doi: 10.1038/s41467-022-30908-1 (PMC9205915; doi:10.1038/s41467-022-30908-1)
Supplement: Supplementary file 1 — Supplementary Information [file 41467_2022_30908_MOESM1_ESM.pdf]

Supplementary Information

Supplementary notes

Inverse probability treatment weighting (IPTW)

IPTW was used to adjust for differences in baseline covariates between pralsetinib and pembrolizumab, and then pralsetinib and pembrolizumab with chemotherapy. This allowed comparison of time to event endpoints between these groups after minimizing imbalances in prognostic variables. The estimand of interest was the ATT, which utilises the ARROW cohort as the target population, i.e., patients enrolled in the comparator study are re-weighted to match the characteristics of ARROW patients. A logistic regression was used to model the probability $P(S|L)$ of having a pralsetinib $S = 1$ at diagnosis conditional on baseline covariates L. Patients were assigned a weight of 1 in the pralsetinib ("treated") arm, while each patient in the comparator arm was assigned the weight corresponding to the odds of being treated, i.e., conditional probability of being treated/(1-conditional probability of being treated). Patients with weights greater than three were removed "trimmed" to prevent a small number of patients from having a disproportionately large effect on the results. Variables in the logistic regression model were – age, sex, ECOG PS, cancer stage, race, time since initial diagnosis, and smoking history. IPTW based on this model yielded sufficient balance across all covariates of interest used in the primary analysis except ECOG PS and race, which were therefore also included as covariates in the Cox model. IPTW modelling was blinded to the study outcomes. Robust standard errors were estimated to account for weighting.

QBA for missing data assumptions about baseline covariates: delta (δ)-based tipping point analysis

Given the non-negligible proportion of missing data (>10%) in baseline ECOG PS, we performed a tipping point-based bias analysis using δ-based shifts under the assumption that ECOG PS was missing not at random (MNAR) for the RWD group, which was larger. For δ adjustments δ was an additive term applied to the ordered logistic regression model for ECOG PS (Y) and $j = \{0, 1, 2, 3, 4\}$ representing $log\frac{p(Y \leq j)}{p(Y > j)}$.

For the adjustments, fixed constant values of δ of 1, 0, -1, -2, -3, -4 or -5 were added to the ordered logistic regression imputation model for ECOG PS. Positive values for δ probabilistically shifted imputed ECOG PS to be more favorable than expected under MAR (i.e., assigning a lower ECOG PS than imputed given other measured covariates) for those missing ECOG PS. Conversely, a negative δ randomly shifted imputed ECOG PS to be poorer than expected under MAR. 20 datasets were multiply imputed for each setting of the δ parameter.

QBA of unmeasured confounding

This analysis examines the effect of unmeasured confounding that would be required to nullify or reverse the conclusions of this study. We assume for interpretability that a hypothetical binary confounder U underlies the residual and/or unmeasured confounding on the estimated treatment effects from this study. By assessing how strong of a confounder U would have to be to nullify or reverse our conclusions, we can measure the robustness of this study. To do this, we calculate the bias B resulting from U as a function of

(1) association of U with the outcome on the risk ratio scale (RR_{UD}), and

(2) imbalance of U between treatment arms on the risk ratio scale (RR_{EU}) as in VanderWeele & Ding, 2017[1].

Because only risk ratios are handled, hazard ratios were converted to approximate risk ratios using the square-root transformation[2]. HRs from multiple imputation reported used here for the bias plots for the worst-case scenario.

QBA of hazard ratio robustness: transformation-threshold delta (δ)-based tipping point analysis

Transformation-threshold tipping point analysis was performed by multiplying the recorded time to event for each outcome by increasing values of a multiplicative constant delta (δ) between 1 and 2 for the reference group (1L pembrolizumab or 1L pembrolizumab with chemotherapy). δ=1 represents the original time to event data, and δ=2 is time to event that is twice as long as the original for each patient. To maintain a

fixed follow-up time, patients were censored if their transformed time to event was greater than the maximum follow-up time in the original data for the reference group. Median time to event and 95% CI were calculated using weighted Kaplan-Meier analysis. Hazard ratios and 95% CI were estimated using a weighted Cox model.

Proportional-hazards assumption diagnostics for the ARROW vs EDM comparisons

The relevant figures in this section are: Supplementary Figure 1: Log-negative-log plot for the comparison with pembrolizumab in 1L, and Supplementary Figure 2: Log-negative-log plot for the comparison with pembrolizumab with chemotherapy in 1L.

Based on the Schoenfeld test, the PH assumption is rejected at the 5% level for the IPTW model for PFS for the pembrolizumab 1L comparison. However, based on the LNL and KM plots, assuming proportional hazards is reasonable.

The PH assumption was not violated for the remaining analyses between pralsetinib and pembrolizumab or those with pembrolizumab with chemotherapy in 1L. This was concluded as the results from the Schoenfeld tests were in agreement with inspection of the LNL and KM plots.

Sensitivity analysis – adjusting for metastases

Pembrolizumab

Following IPTW, Supplementary Table 1 shows that balance was achieved for some covariates used for adjustment though age, sex, smoking history, and race are still imbalanced. Sum of total metastases is highly imbalanced, though metastases-related variables are suspected to be unreliable.

All estimates in Supplementary Table 2 are significantly in favour of pralsetinib. This is the same result as that in the main analysis with most of the magnitudes being broadly similar. The discrepancies may be explained by the residual imbalance in this sensitivity analysis. Altogether this suggests that the inclusion of Metastases (categorical) in the confounder set likely does not have a notable impact on the results in the main analysis.

See also: Supplementary Figure 3: Kaplan-Meier estimates using IPTW for (A) TTD, (B) OS, and (C) PFS comparing pralsetinib with pembrolizumab in 1L; ESS=109 for the pralsetinib cohort, and ESS=71 for the pembrolizumab cohort; Metastases was explicitly adjusted for during balancing.

Based on the Schoenfeld test, the PH assumption is rejected at the 5% level for all the models for TTD and PFS. However, the corresponding LNL plots for these models suggest that any violation is likely mild for PFS and moderate for TTD. See Supplementary Figure 4.

The PH assumption was not violated for the remaining analyses between pralsetinib and pembrolizumab in 1L. This was concluded as the results from the Schoenfeld tests were in agreement with inspection of the LNL and KM plots.

Pembrolizumab with chemotherapy

The results in Supplementary Table 3 shows that balance was achieved among many of the covariates used for adjustment except smoking history and race are moderately imbalanced, and stage is slightly imbalanced. Metastases-related variables are balanced, except for sum of total metastases

All estimates in Supplementary Table 4 are significantly in favour of pralsetinib. This is the same result as in the main analysis. The discrepancies in magnitude might be attributable to the residual imbalances. Altogether this suggests that the inclusion of metastases in the confounder set likely does not have a notable impact on the results in the main analysis.

See Supplementary Figure 5: Kaplan-Meier estimates using IPTW for (A) TTD, (B) OS, and (C) PFS comparing pralsetinib and pembrolizumab with chemotherapy in 1L; ESS=109 for the pralsetinib cohort, and ESS=116 for the pembrolizumab with chemotherapy cohort; Metastases was explicitly adjusted for during balancing.

The PH assumption was not found to be violated for any analyses between pralsetinib and pembrolizumab with chemotherapy in 1L. This was concluded as the results from the Schoenfeld tests were all in agreement with inspection of the LNL and KM plots. See Supplementary Figure 6: Log-negative-log plot for the comparison with pembrolizumab with chemotherapy in 1L.

CGDB RET fusion-positive analysis

1L BAT regimens

The specific regimens and counts are:

- Pembrolizumab with chemotherapy (N=5)
- Bevacizumab, Carboplatin, Pemetrexed (N=3)
- Atezolizumab (N=1)
- Carboplatin, Pemetrexed (N=1)

CGDB RET fusion-positive cohort eligibility criteria

Key inclusion and exclusion criteria are presented below. For the Flatiron CGDB data, "last follow-up" is defined as the date of the last available visit, lab, treatment, or medication administration (last electronic health record activity). Patients in the CGDB were followed up until a cut-off date of 2020 June 30 .

Inclusion criteria

1. Patients must have unresectable locally advanced or metastatic NSCLC
    - ARROW patients must have a RET-fusion positive tissue sample
    - Flatiron patients must have a negative RET-fusion test from FMI report prior to the treatment line start
2. Flatiron patients must have FMI genetic testing conducted no later than 30 days after treatment line initiation
    - Addresses concerns about possible immortal time bias due to the timing of RET testing
3. Patient has an ECOG of 0 or 1
    - The ARROW data has at most one subject with ECOG > 1. Thus, if CGDB patients with ECOG > 1 are included, the non-overlap between the two datasets becomes an issue that cannot be solved by statistical weighting methods since we can only adjust for ECOG values common in both arms
4. Must be aged ≥ 18 years of age at the initiation of first line systemic therapy.
5. Specimen collection date no later than 1 month after initiation of current line.

6. Follow-up (observation period) must be a minimum of three months from the advanced diagnosis date, or until date of death, if death occurred less than three months from advanced diagnosis date
7. Histology must be non-squamous
    - For each indirect comparison, the ARROW data has at most two patients with squamous histology

Exclusion criteria

1. For CGDB, patients with > 90-day gap between advanced diagnosis and first visit or medication administration or >60-day gap between FMI report and first visit or medicare administration were excluded in accordance with best practices
2. Patients in the CGDB must not have had pralsetinib or selpercatinib or clinical study drugs in any line
3. Patient has another known driver mutation (EGFR, ALK, ROS1 or BRAF) at index date
4. Index date less than 6 months prior to the CGDB cut-off date
5. Previously treated with a selective RET inhibitor
6. For stage at initial diagnosis and smoking status, patients must not have either missing entries or have entries labelled not reported.

Results

Multiple balancing strategies were considered for the comparisons between the pralsetinib and CGDB RET+ 1L BAT cohort but due to sample size restrictions (n=10 patients in 1L BAT), results from the naïve unadjusted comparison were found.

See Supplementary Table 5: Baseline characteristics of the 1L ARROW trial participants given pralsetinib and 1L CGDB cohort given the best available therapy without adjustment; variables with SMD<0.1 are considered balanced, Supplementary Table 6: Median endpoints of the 1L ARROW trial participants (including the safety population) and Flatiron Health cohort without propensity score weighting, Supplementary Figure 7: Kaplan-Meier estimates for overall survival in the ARROW trial (including the safety population) and Flatiron Health cohort for first line therapy; N=116 for the pralsetinib cohort, and N=10 for the BAT cohort, Supplementary Figure 8: Kaplan-Meier estimates for progression free survival in the ARROW trial (including the safety population) and Flatiron Health cohort for first line therapy; N=116 for the pralsetinib cohort, and N=10 for the BAT cohort, and Supplementary Figure 9: Kaplan-Meier estimates for time-to-treatment discontinuation in the ARROW trial (including the safety population) and Flatiron Health cohort for first line therapy; N=116 for the pralsetinib cohort, and N=10 for the BAT cohort.

QBA of hazard ratio robustness

We also performed an analysis looking into the comparison between the ARROW-adjusted EDM vs KEYNOTE using matching-adjusted indirect comparisons (MAICs).

We found that after balancing the baseline patient characteristics (included key ones like age, sex, ECOG, smoking), the OS curve only shifted by a very small amount. i.e. the median OS barely changed. This suggests that any differences in OS between the ARROW-adjusted EDM and KEYNOTE cohorts are largely not due to differences in patient characteristics. Thus, we felt that the results of this QBA could be used to claim that our results were robust.

See Supplementary Table 7: Tipping point analysis for pralsetinib versus pembrolizumab comparison. Median values represent median time to event values in the pembrolizumab group. The median OS at the transformation threshold is 34.58 months, and the HR is 0.53 (0.29-0.96) with a Cox p-value of 0.038. *values represent loss of statistical significance. All statistical tests were two-sided at a 5% significance level.

Variable definitions

Variable definitions for baseline covariates included in this study.

- Age at index date (years) – Categorized as <65 or ≥65 years of age
- Sex – Male or female
- Race – White, Other (Asian, African America/Black, or Latino/Hispanic) or Unknown
- Cancer stage at diagnosis – Categorized as stage I-III or IV
- Tumor histology – non-squamous, squamous or unknown
- ECOG PS – Categorized as 0, 1 or 2+
    - Baseline ECOG PS was measured between 30 days prior to and/or 7 days after index date
- Time from initial diagnosis to index date (months)
- Metastases (categorical) – identified using ICD-9/10 codes. Categorized as present or absent.
- Sum of metastases – Defined as sum of metastasis sites for given patient
- Metastases (collapsed) – Defined as presence of brain/CNS metastasis for 'Brain/CNS', 'Other' if lung, liver, other, or bone metastasis, and 'None' if no defined metastasis for any groups.

In the real-world dataset, dates of death were not recorded, and only month and year were available. Therefore, the 15th day of the month was used to derive overall survival.

Supplementary figures

Supplementary Figure 1: Log-negative-log plot for the comparison with pembrolizumab in 1L; for all three plots, ESS=109 for the pralsetinib cohort, and ESS=115 for the pembrolizumab cohort.

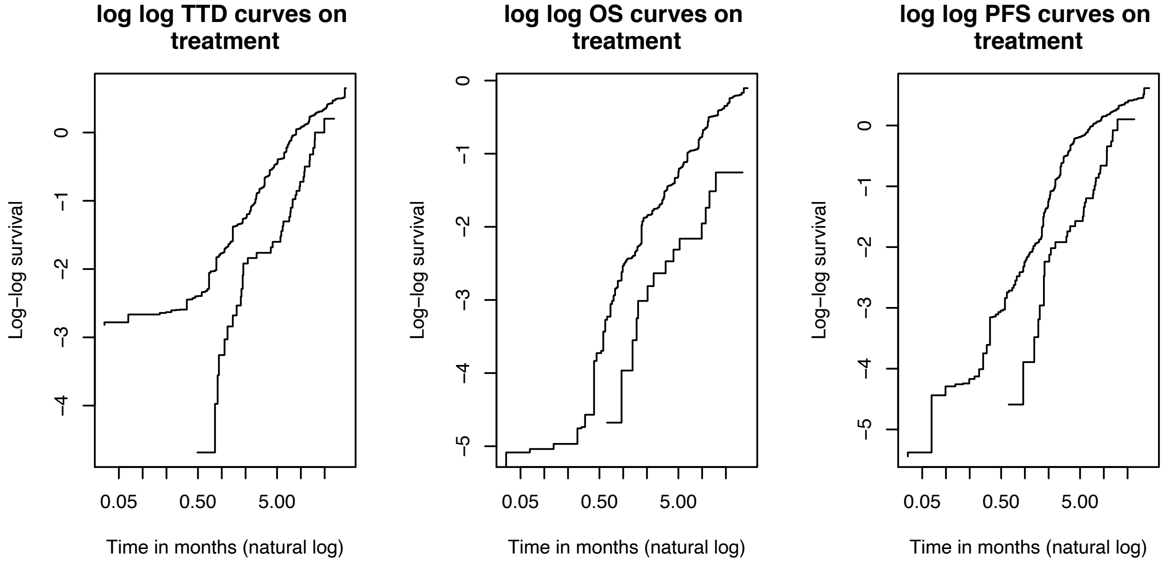

Supplementary Figure 2: Log-negative-log plot for the comparison with pembrolizumab with chemotherapy in 1L; for all three plots, ESS=109 for the pralsetinib cohort, and ESS=217 for the pembrolizumab with chemotherapy cohort.

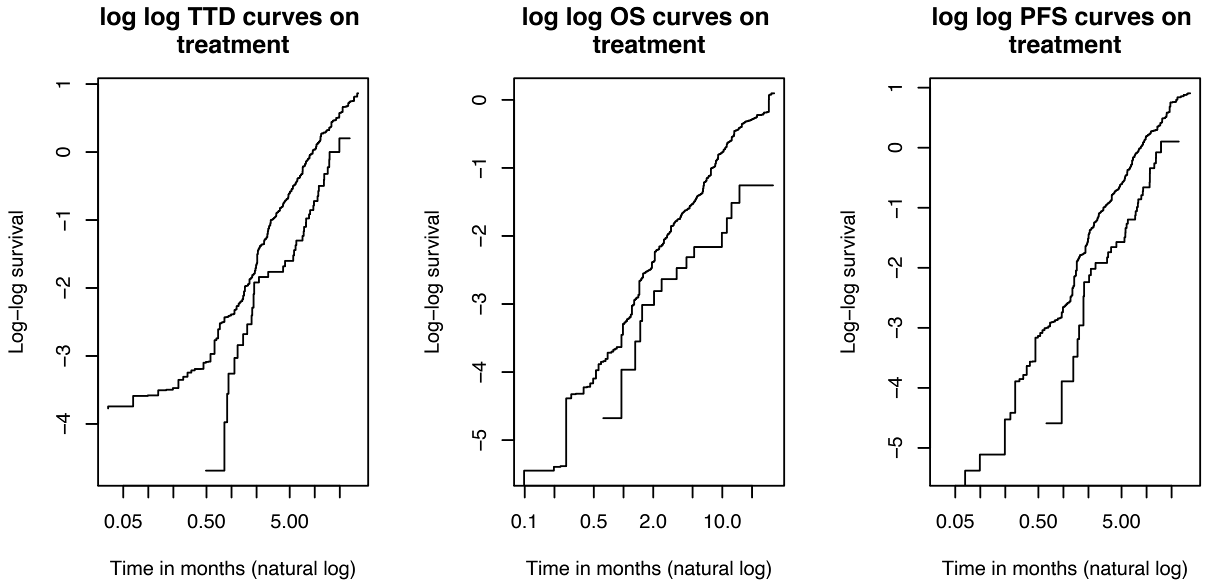

Supplementary Figure 3: Kaplan-Meier estimates using IPTW for (A) TTD, (B) OS, and (C) PFS comparing pralsetinib with pembrolizumab in 1L; ESS=109 for the pralsetinib cohort, and ESS=71 for the pembrolizumab cohort; Metastases was explicitly adjusted for during balancing

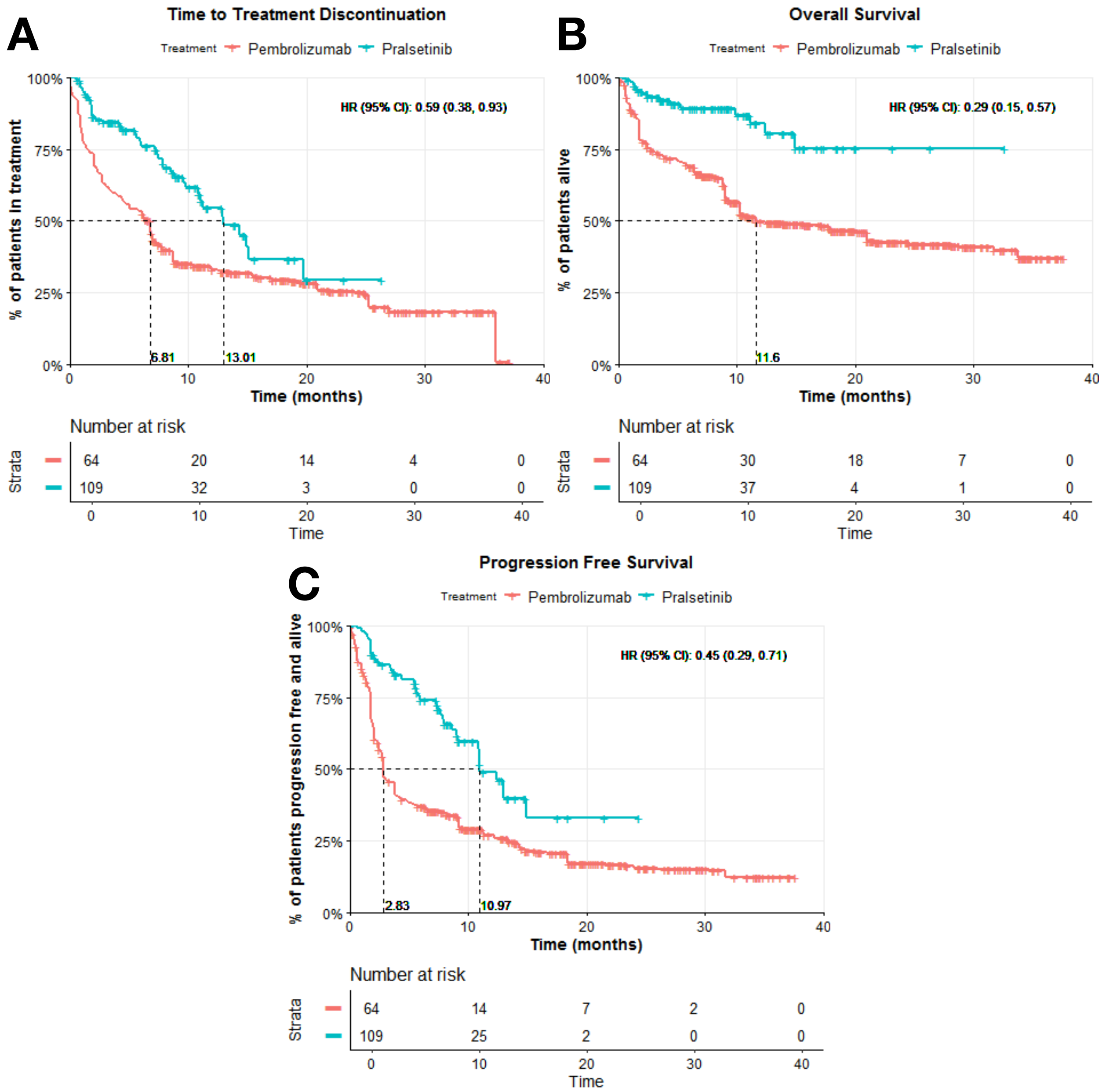

Supplementary Figure 4: Log-negative-log plot for the comparison between pralsetinib and pembrolizumab in 1L; for all three plots, ESS=109 for the pralsetinib cohort, and ESS=71 for the pembrolizumab cohort

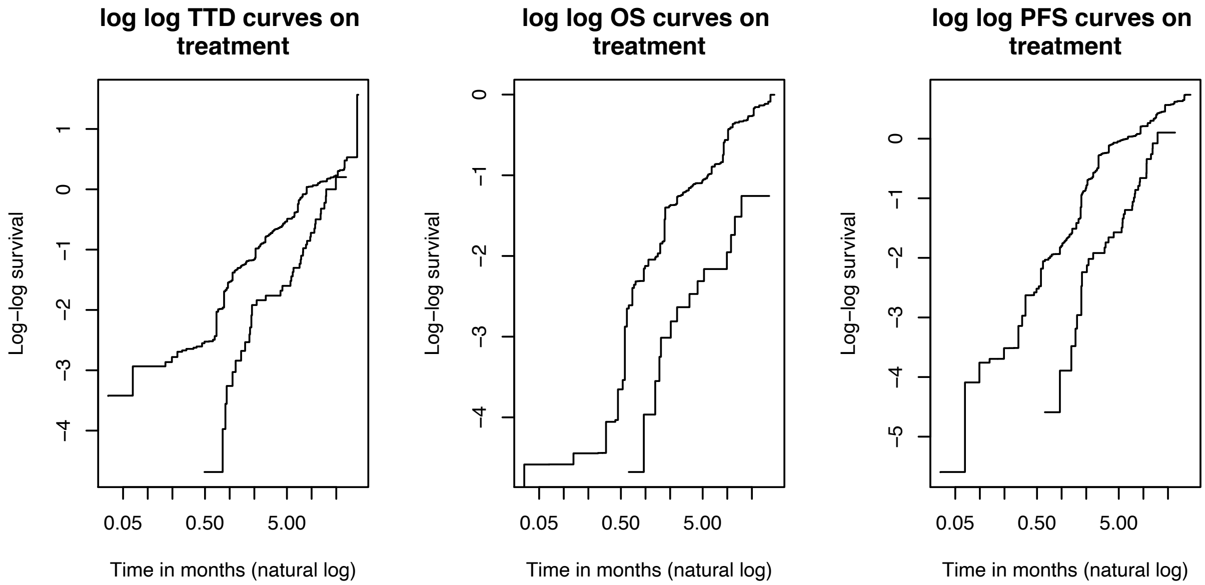

Supplementary Figure 5: Kaplan-Meier estimates using IPTW for (A) TTD, (B) OS, and (C) PFS comparing pralsetinib and pembrolizumab with chemotherapy in 1L; ESS=109 for the pralsetinib cohort, and ESS=116 for the pembrolizumab with chemotherapy cohort; Metastases was explicitly adjusted for during balancing

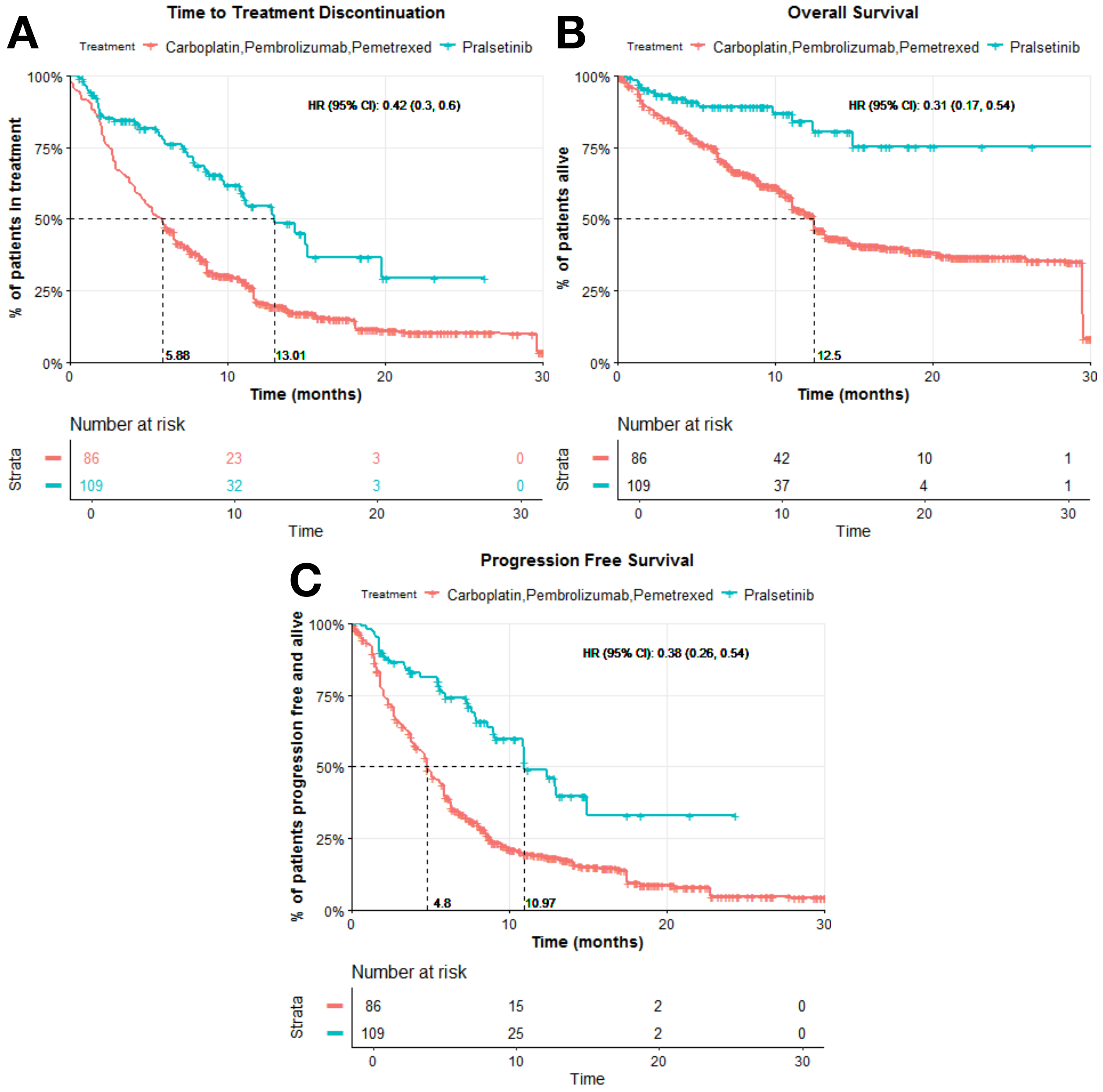

Supplementary Figure 6: Log-negative-log plot for the comparison with pembrolizumab with chemotherapy in 1L; for all three plots, ESS=109 for the pralsetinib cohort, and ESS=116 for the pembrolizumab with chemotherapy cohort

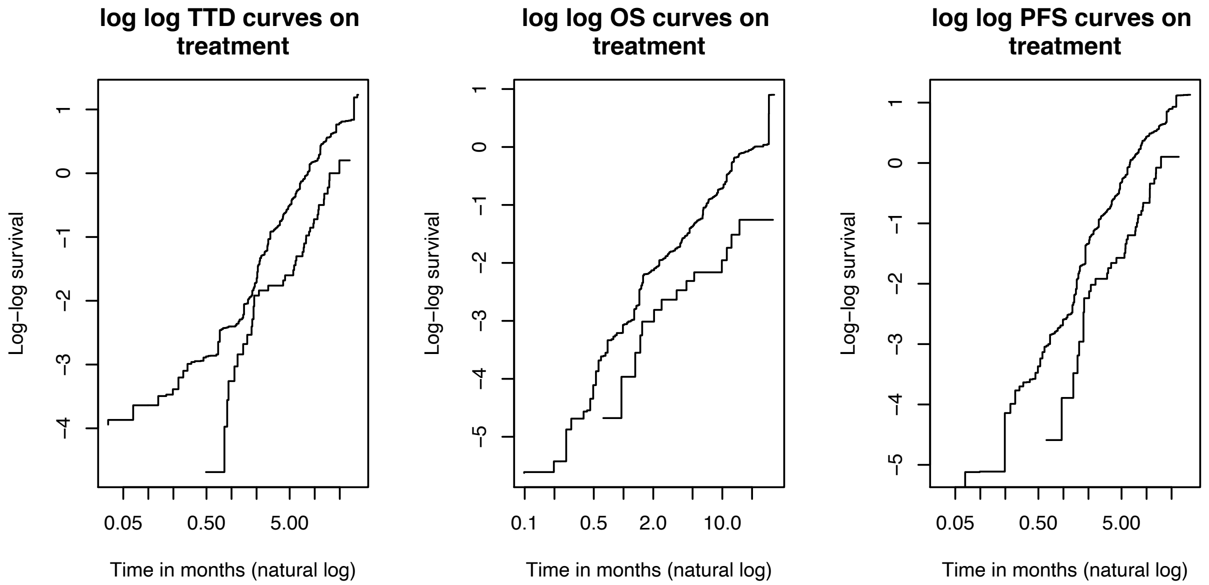

Supplementary Figure 7: Kaplan-Meier estimates for overall survival in the ARROW trial (including the safety population) and Flatiron Health cohort for first line therapy; N=116 for the pralsetinib cohort, and N=10 for the BAT cohort

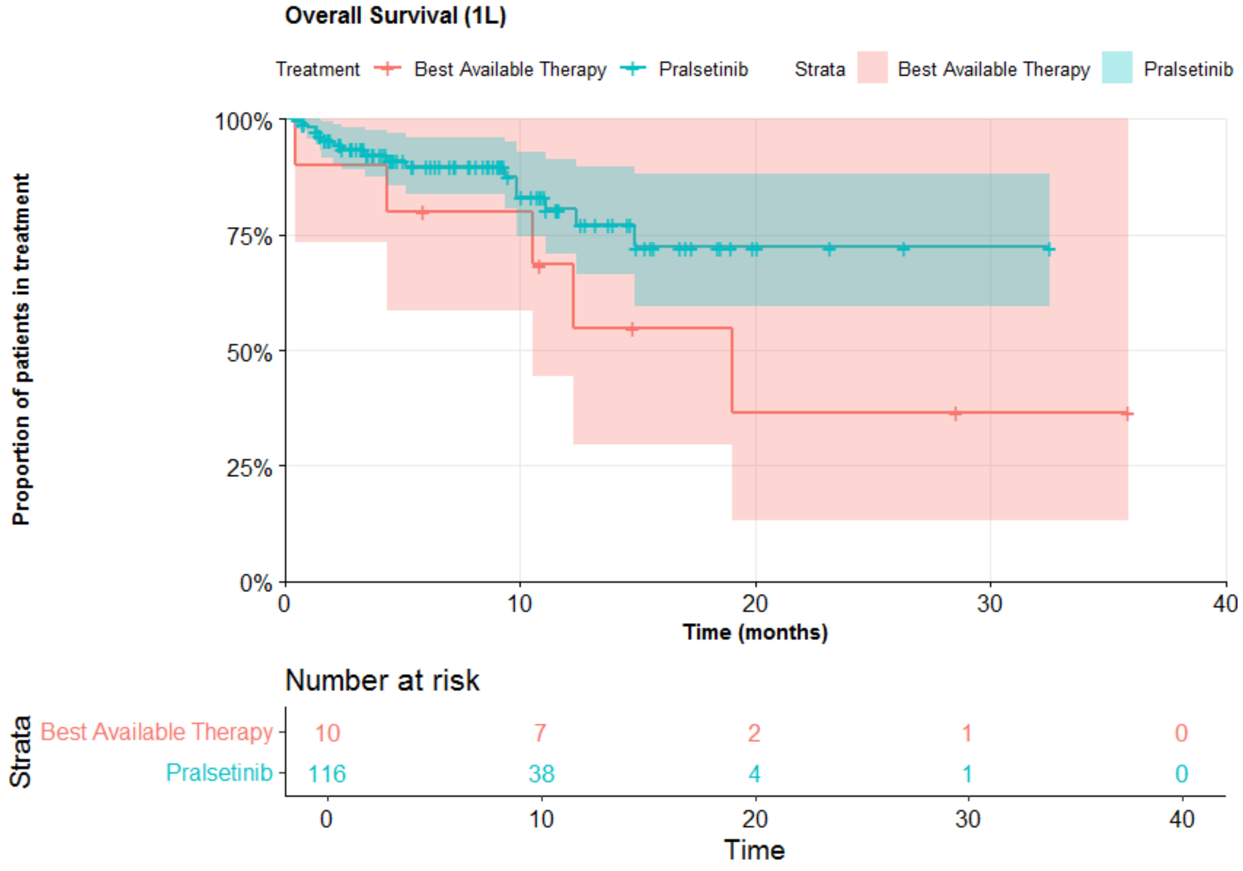

Supplementary Figure 8: Kaplan-Meier estimates for progression free survival in the ARROW trial (including the safety population) and Flatiron Health cohort for first line therapy; N=116 for the pralsetinib cohort, and N=10 for the BAT cohort

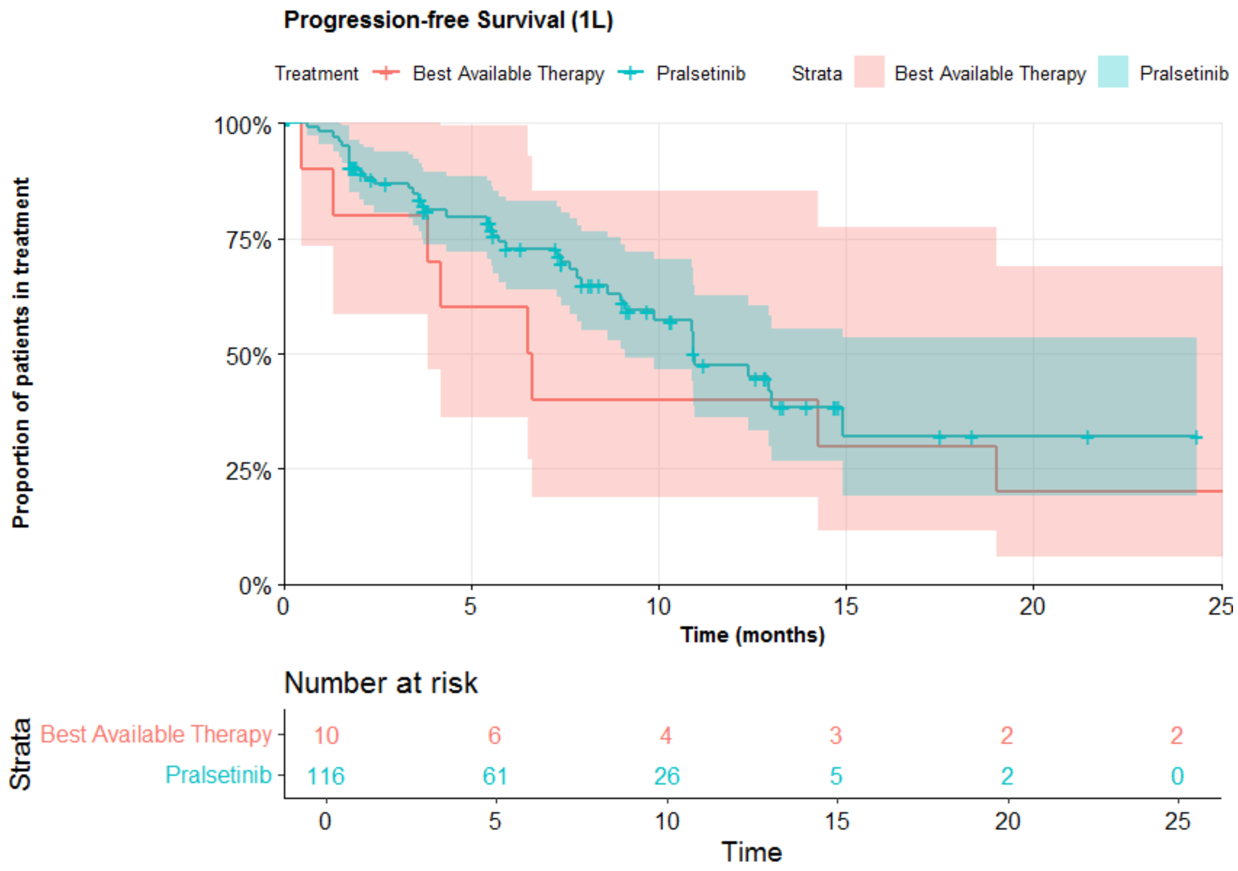

Supplementary Figure 9: Kaplan-Meier estimates for time-to-treatment discontinuation in the ARROW trial (including the safety population) and Flatiron Health cohort for first line therapy; N=116 for the pralsetinib cohort, and N=10 for the BAT cohort

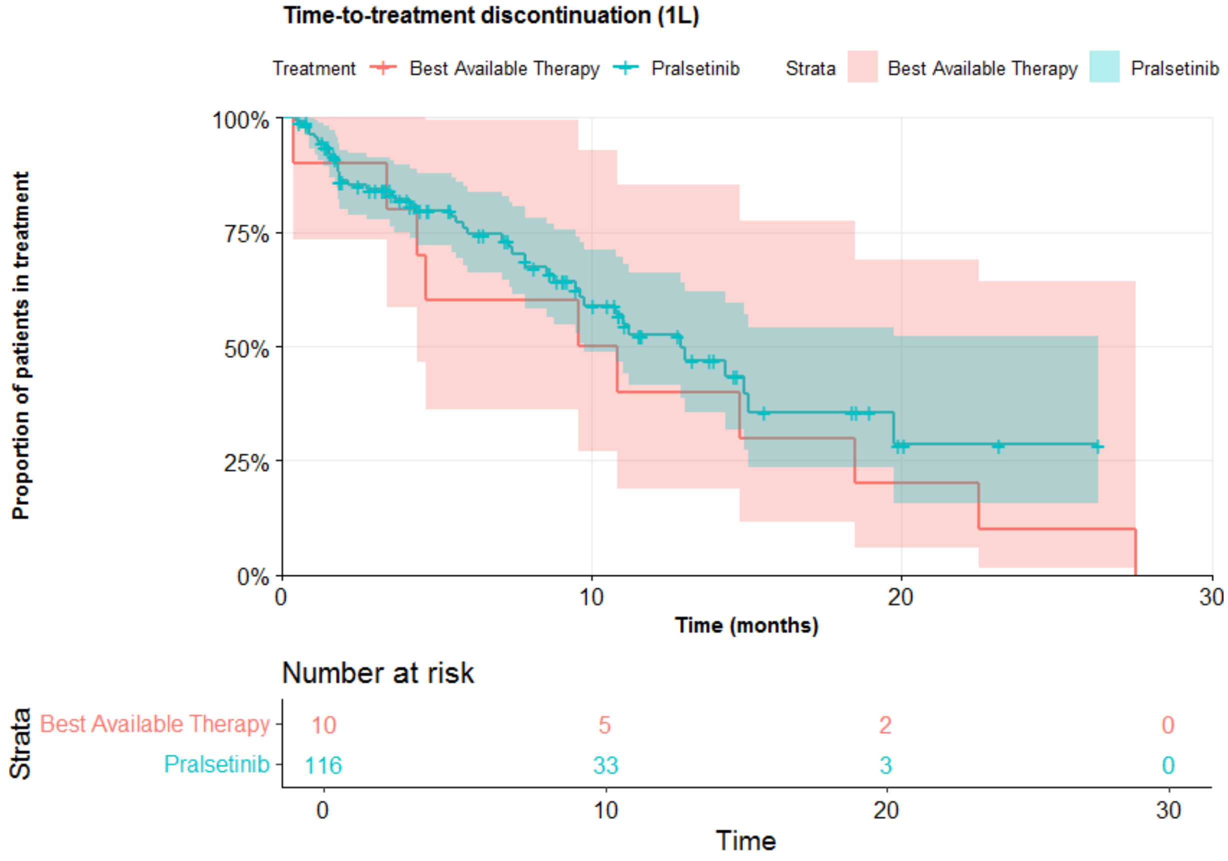

Supplementary tables

Supplementary Table 1: Baseline characteristics of the ARROW trial participants given pralsetinib and Flatiron EDM cohort given pembrolizumab in 1L following IPTW; variables with SMD<0.1 are considered balanced

	Level	Pembrolizumab	Pralsetinib	SMD	Adjusted
**Age (%)**	< 65	47.2	59.6	0.25	Y
	>= 65	52.8	40.4		
**Sex (%)**	F	44.5	54.1	0.193	Y
	M	55.5	45.9		
**Smoking history at baseline (%)**	History of smoking	63.5	39.4	0.497	Y
	No history of smoking	36.5	60.6		
**ECOG (%)**	0	33.7	31.2	0.054	Y
	1	66.3	68.8		
**Time from initial diagnosis to first dose (months) (median [IQR])**		1.45 [0.95, 2.49]	1.74 [1.25, 2.30]	0.085	Y
**Stage at initial diagnosis (%)**	STAGE I, II, or III	17	15.6	0.039	Y
	STAGE IV	83	84.4		
**Race (%)**	White	64.9	49.5	0.428	Y
	Other	25.3	45		
	Unknown	9.8	5.5		
**Metastases (%)**	Isolated brain/CNS site	36.8	27.5	0.229	Y
	None	3.2	1.8		
	Other	60	70.6		
**Brain/CNS metastasis only (%)**	0	63.2	72.5	0.2	N
	1	36.8	27.5		
**Liver metastasis only (%)**	0	93.1	86.2	0.227	N
	1	6.9	13.8		

Supplementary Table 2: Estimates of hazard ratios, restricted mean survival time differences, and their 95% confidence intervals for TTD, OS, and PFS comparing pralsetinib with pembrolizumab in 1L; an asterisk indicates possible violation of the PH assumption based on the Schoenfeld test at the 5% level; Metastases (categorical) was included in the confounder set

Effect	HR	95% CI
TTD Weighted PH*	0.59	[0.38, 0.93]
OS Weighted PH	0.29	[0.15, 0.57]
PFS Weighted PH*	0.45	[0.29, 0.71]

Supplementary Table 3: Baseline characteristics of the ARROW trial participants given pralsetinib and Flatiron EDM cohort given pembrolizumab with chemotherapy in 1L following IPTW; variables with SMD<0.1 are considered balanced

	Level	Pembrolizumab with chemotherapy	Pralsetinib	SMD	Adjusted
**Age (%)**	< 65	56	59.6	0.073	Y
	>= 65	44	40.4		
**Sex (%)**	F	50.6	54.1	0.07	Y
	M	49.4	45.9		
**Smoking history at baseline (%)**	History of smoking	49	39.4	0.193	Y
	No history of smoking	51	60.6		
**ECOG (%)**	0	32.4	31.2	0.025	Y
	1	67.6	68.8		
**Time from initial diagnosis to first dose (months) (median [IQR])**		1.15 [0.82, 1.74]	1.74 [1.25, 2.30]	0.10	Y
**Stage at initial diagnosis (%)**	STAGE I, II, or III	11	15.6	0.135	Y
	STAGE IV	89	84.4		
**Race (%)**	White	59.9	49.5	0.283	Y
	Other	31.6	45		
	Unknown	8.5	5.5		
**Metastases (%)**	Isolated brain/CNS site	30.7	27.5	0.083	Y
	None	2.4	1.8		
	Other	66.9	70.6		
**Brain/CNS metastasis only (%)**	0	69.3	72.5	0.07	N
	1	30.7	27.5		
**Liver metastasis only (%)**	0	88.1	86.2	0.055	N
	1	11.9	13.8		

Supplementary Table 4: Estimates of hazard ratios, restricted mean survival time differences, and their 95% confidence intervals for time-to-treatment discontinuation, overall survival, and progression-free survival comparing pralsetinib with pembrolizumab with chemotherapy in 1L; Metastases was included in the confounder set

Effect	HR	95% CI
TTD Weighted PH	0.42	[0.3, 0.6]
OS Weighted PH	0.31	[0.17, 0.54]
PFS Weighted PH	0.38	[0.26, 0.54]

Supplementary Table 5: Baseline characteristics of the 1L ARROW trial participants given pralsetinib and 1L CGDB cohort given the best available therapy without adjustment; variables with SMD<0.1 are considered balanced

Baseline characteristics	Best Available Therapy	Pralsetinib	SMD
**Sample size – n**	10	116	--
**Age ≥ 65 – n (%)**	5 (50.0)	49 (42.2)	0.156
**Male – n (%)**	2 (20.0)	55 (47.4)	0.606
**Stage IV – n (%)**	7 (70.0)	95 (81.9)	0.281
**Smoking status – n (%)**			
**History of smoking**	4 (40.0)	45(38.8)	
**No history of smoking**	6 (60.0)	68(58.6)	0.23
**Unknown**	0 (0.0)	3 (2.6)	
**ECOG – n (%)**			
**0**	5 (50.0)	35 (30.2)	
**1**	3 (30.0)	80 (69.0)	1.017
**2**	0 (0.0)	1 (0.9)	
**Missing**	2 (20.0)	0 (0.0)	
**Non-squamous histology – n (%)**	10 (100.0)	115 (99.3)	0.132
**Time since diagnosis – median (IQR)**	1.50 (0.77, 8.77)	1.76 (1.25, 2.51)	0.069
**Metastases: Brain/CNS site – n (%)**	1 (10.0)	31 (26.7)	0.442
**Metastases: Bone – n (%)**	2 (20.0)	44 (37.9)	0.403
**Metastases: Liver – n (%)**	3 (30.0)	16 (13.8)	0.4
**Metastases: Lung – n (%)**	1 (10.0)	28 (24.1)	0.383
**Metastases: Other – n (%)**	4 (40.0)	86 (74.1)	0.735
**Race – n (%)**			
**Other**	2 (20.0)	53 (45.7)	
**Unknown**	2 (20.0)	6 (5.2)	0.664
**White**	6 (60.0)	57 (49.1)	
**Year of line of therapy initiation – n (%)**			
**2015**	1 (10.0)	0 (0.0)	
**2016**	2 (20.0)	0 (0.0)	
**2017**	3 (30.0)	0 (0.0)	
**2018**	2 (20.0)	0 (0.0)	--
**2019**	2 (20.0)	0 (0.0)	
**2017-2019**	0 (0.0)	116 (100.0)	

Acronyms: ECOG: Eastern Cooperative Oncology Group, IQR: Interquartile range, SMD: standardized mean difference

Supplementary Table 6: Median endpoints of the 1L ARROW trial participants (including the safety population) and Flatiron Health cohort without propensity score weighting

**1L endpoint**	**Best available therapy**	**Pralsetinib**
**Sample size**	10	116
**OS – median (95% CI)**	19.0 (10.6, NA)	Not reached
**PFS – median (95% CI)**	6.6 (3.9, NA)	11.0 (9.1, NA)
**TTD – median (95% CI)**	10.2 (4.4, NA)	12.9 (9.8, NA)

Upper confidence interval values = NA if the calculation did not yield a finite upper bound

**RWD**	**Effect**	**HR**	**95% CI**
CGDB RET+ 1L BAT	TTD Unadjusted PH	0.71	[0.31, 1.59]
CGDB RET+ 1L BAT	OS Unadjusted PH	0.45	[0.16, 1.25]
CGDB RET+ 1L BAT	PFS Unadjusted PH	0.71	[0.32, 1.55]

Supplementary Table 7: Tipping point analysis for pralsetinib versus pembrolizumab comparison. Median values represent median time to event values in the pembrolizumab group. The median OS at the transformation threshold is 34.58 months, and the HR is 0.53 (0.29-0.96) with a Cox p-value of 0.038. *values represent loss of statistical significance. All statistical tests were two-sided at a 5% significance level.

Delta	Median OS	Log-rank p-value	HR	Cox p-value
1	19.17 (10.22-NA)	<0.01	0.35 (0.19-0.64)	<0.01
1.1	21.08 (11.25-NA)	<0.01	0.37 (0.20-0.68)	<0.01
1.2	23.00 (12.27-NA)	<0.01	0.40 (0.22-0.72)	<0.01
1.3	24.92 (13.29-NA)	<0.01	0.42 (0.23-0.77)	<0.01
1.4	26.83 (14.31-NA)	<0.01	0.45 (0.24-0.81)	<0.01
1.5	28.75 (15.34-NA)	<0.01	0.47 (0.26-0.86)	0.014
1.6	30.67 (16.36-NA)	0.012	0.49 (0.27-0.90)	0.022
1.7	32.58 (17.38-NA)	0.019	0.53 (0.29-0.96)	0.038
1.8	34.50 (18.40-NA)	0.029	0.56 (0.31-1.01)*	0.056*
1.9	36.42 (19.43-NA)	0.042	0.58 (0.32-1.06)*	0.076*
2	NA (20.45-NA)*	0.058*	0.61 (0.33-1.12)*	0.11*

Delta	Median PFS	Log-rank p-value	HR	Cox p-value
1	3.52 (2.76-6.58)	<0.01	0.45 (0.31-0.66)	<0.01
1.1	3.87 (3.04-7.23)	<0.01	0.47 (0.32-0.69)	<0.01
1.2	4.22 (3.31-7.89)	<0.01	0.50 (0.34-0.74)	<0.01
1.3	4.57 (3.59-8.55)	0.022	0.53 (0.36-0.78)	<0.01
1.4	4.92 (3.87-9.21)*	0.051*	0.55 (0.37-0.82)	<0.01
1.5	5.28 (4.14-9.86)*	0.098*	0.58 (0.39-0.86)	<0.01
1.6	5.63 (4.42-10.52)*	0.167*	0.60 (0.40-0.90)	0.013
1.7	5.98 (4.69-11.18)*	0.265*	0.62 (0.41-0.93)	0.02
1.8	6.33 (4.97-11.84)*	0.38*	0.65 (0.43-0.97)	0.036
1.9	6.68 (5.25-12.49)*	0.53*	0.67 (0.45-1.01)*	0.054*
2	7.04 (5.52-13.15)*	0.697*	0.70 (0.47-1.06)*	0.089*

Delta	Median TTD	Log-rank p-value	HR	Cox p-value
1	4.04 (2.66-6.58)	<0.01	0.44 (0.30-0.63)	<0.01
1.1	4.45 (2.93-7.23)	<0.01	0.46 (0.32-0.67)	<0.01
1.2	4.85 (3.20-7.89)	0.017	0.49 (0.34-0.71)	<0.01
1.3	5.26 (3.46-8.55)	0.041	0.52 (0.35-0.75)	<0.01
1.4	5.66 (3.73-9.21)*	0.08*	0.54 (0.37-0.79)	<0.01
1.5	6.07 (3.99-9.86)*	0.148*	0.57 (0.39-0.83)	<0.01
1.6	6.47 (4.26-10.52)*	0.243*	0.60 (0.41-0.87)	<0.01
1.7	6.87 (4.53-11.18)*	0.347*	0.62 (0.42-0.91)	0.015
1.8	7.28 (4.79-11.84)*	0.478*	0.65 (0.44-0.96)	0.029
1.9	7.68 (5.06-12.49)*	0.628*	0.67 (0.46-1.00)	0.047
2	8.09 (5.33-13.15)*	0.79*	0.69 (0.47-1.03)*	0.068*

Supplementary Table 8: Tipping point analysis for pralsetinib versus pembrolizumab with chemotherapy comparison. Median values represent median time to event values in the pembrolizumab with chemotherapy group. The median OS at the transformation threshold is 25.20 months, and the HR is 0.56 (0.32-0.99) with a Cox p-value of 0.046. *values represent loss of statistical significance. All statistical tests were two-sided at a 5% significance level.

Delta	Median OS	Log-rank p-value	HR	Cox p-value
1	15.75 (12.46-31.36)	<0.01	0.37 (0.21-0.65)	<0.01
1.1	17.32 (13.71-NA)	<0.01	0.40 (0.23-0.70)	<0.01
1.2	18.90 (14.95-NA)	<0.01	0.43 (0.25-0.76)	<0.01
1.3	20.47 (16.20-NA)	<0.01	0.47 (0.27-0.81)	<0.01
1.4	22.05 (17.44-NA)	<0.01	0.50 (0.28-0.87)	0.015
1.5	23.62 (18.69-NA)	<0.01	0.53 (0.30-0.93)	0.027
1.6	25.20 (19.94-NA)	<0.01	0.56 (0.32-0.99)	0.046
1.7	26.77 (21.18-NA)	<0.01	0.60 (0.34-1.06)*	0.077*
1.8	28.35 (22.43-NA)	0.015	0.64 (0.36-1.12)*	0.118*
1.9	29.92 (23.67-NA)	0.025	0.67 (0.38-1.18)*	0.163*
2	31.50 (24.92-NA)	0.039	0.70 (0.40-1.24)*	0.225*

Delta	Median PFS	Log-rank p-value	HR	Cox p-value
1	6.08 (5.33-6.94)	<0.01	0.51 (0.36-0.71)	<0.01
1.1	6.69 (5.86-7.63)	<0.01	0.55 (0.39-0.77)	<0.01
1.2	7.30 (6.39-8.32)	<0.01	0.60 (0.43-0.84)	<0.01
1.3	7.91 (6.92-9.02)	<0.01	0.65 (0.46-0.91)	0.012
1.4	8.51 (7.46-9.71)	0.017	0.70 (0.50-0.99)	0.044
1.5	9.12 (7.99-10.41)	0.048	0.75 (0.54-1.06)*	0.107*
1.6	9.73 (8.52-11.10)*	0.109*	0.81 (0.57-1.15)*	0.234*
1.7	10.34 (9.05-11.79)*	0.213*	0.86 (0.61-1.22)*	0.411*
1.8	10.95 (9.59-12.49)*	0.365*	0.90 (0.64-1.28)*	0.572*
1.9	11.56 (10.12-13.18)*	0.565*	0.96 (0.67-1.37)*	0.821*
2	12.16 (10.65-13.87)*	0.806*	1.02 (0.71-1.46)*	0.908*

Delta	Median TTD	Log-rank p-value	HR	Cox p-value
1	6.64 (5.26-7.82)	<0.01	0.50 (0.36-0.70)	<0.01
1.1	7.31 (5.79-8.61)	<0.01	0.55 (0.39-0.76)	<0.01
1.2	7.97 (6.31-9.39)	<0.01	0.59 (0.42-0.82)	<0.01
1.3	8.63 (6.84-10.17)	<0.01	0.64 (0.46-0.89)	<0.01
1.4	9.30 (7.36-10.95)	0.013	0.68 (0.49-0.96)	0.026
1.5	9.96 (7.89-11.74)	0.034	0.73 (0.52-1.02)*	0.064*
1.6	10.63 (8.42-12.52)*	0.078*	0.77 (0.55-1.08)*	0.135*
1.7	11.29 (8.94-13.30)*	0.154*	0.82 (0.58-1.16)*	0.258*
1.8	11.95 (9.47-14.08)*	0.267*	0.87 (0.61-1.22)*	0.416*
1.9	12.62 (9.99-14.87)*	0.415*	0.91 (0.64-1.28)*	0.585*
2	13.28 (10.52-15.65)*	0.602*	0.96 (0.68-1.36)*	0.819*

Supplementary references

1. VanderWeele, T. J. & Ding, P. Sensitivity Analysis in Observational Research: Introducing the E-Value. *Ann. Intern. Med.* **167**, 268 (2017).
2. VanderWeele, T. J. On a Square-Root Transformation of the Odds Ratio for a Common Outcome: *Epidemiology* **28**, e58–e60 (2017).
